# Continuous Evolution of Statistical Estimators for Optimal Decision-Making

**DOI:** 10.1371/journal.pone.0037547

**Published:** 2012-06-25

**Authors:** Ian Saunders, Sethu Vijayakumar

**Affiliations:** Institute of Perception, Action and Behaviour, School of Informatics, University of Edinburgh, Edinburgh, United Kingdom; Bielefeld University, Germany

## Abstract

In many everyday situations, humans must make precise decisions in the presence of uncertain sensory information. For example, when asked to combine information from multiple sources we often assign greater weight to the more reliable information. It has been proposed that statistical-optimality often observed in human perception and decision-making requires that humans have access to the uncertainty of both their senses and their decisions. However, the mechanisms underlying the processes of uncertainty estimation remain largely unexplored. In this paper we introduce a novel visual tracking experiment that requires subjects to continuously report their evolving perception of the mean and uncertainty of noisy visual cues over time. We show that subjects accumulate sensory information over the course of a trial to form a continuous estimate of the mean, hindered only by natural kinematic constraints (sensorimotor latency etc.). Furthermore, subjects have access to a measure of their continuous objective uncertainty, rapidly acquired from sensory information available within a trial, but limited by natural kinematic constraints and a conservative margin for error. Our results provide the first direct evidence of the continuous mean and uncertainty estimation mechanisms in humans that may underlie optimal decision making.

## Introduction

Uncertainty is a fundamental property of the world, as any avid butterfly collector will attest. To anticipate the fluttering flight of *papilionoidea*, one must wait patiently, accumulating evidence about the underlying statistics of its rapid and unpredictable movements. Success is only achieved when one is prepared with a large enough net to accommodate the variability in both the butterfly’s trajectory and the movement of one’s arm.

To handle the inevitable uncertainty in the world, people make decisions based on previous experience, as well as statistical information acquired directly from stimuli. For example, the statistics of the environment govern our perceptions and our decision making processes when we reach for targets [1], [2], interpret visual scenes [3]–[5] and combine multiple sensory modalities [6]–[9]. This growing body of psychophysical experiments supports the proposition that some aspects of perception are statistically-optimal, in the sense that decisions made are often quantitatively indistinguishable from a *maximum-likelihood* ideal observer (although some studies are inconsistent with this theory [10]–[13]). To achieve optimality when combining multiple sensory cues, the nervous system requires an estimate of the reliabiliy of the sensory information [3], [14]. However, despite its fundamental importance to the theory, the question of *how* humans gather the relevant statistical information to make their optimal decisions remains largely unexplored [15].

The theory of statistical optimality in the brain relies crucially on the fact that humans must somehow accumulate statistical information from unpredictable stimuli. For example they may need to estimate not only the *mean*, but the expected variability in this estimate of the mean (or their *confidence*). Recently, it was shown that humans are not only able to predict the position of objects moving along random or noisy trajectories, but also that they are able to report a level of confidence in this prediction [16]. This is not a uniquely human capacity: rats are also capable of uncertainty-based decisions [17]. It has been shown that *subjective* perception of uncertainty is closely related to the *objective* uncertainty (the measured variability in performance) [15], indicating that subjects are, indeed, acutely aware of the uncertainty in their decisions.

The forced-choice paradigm is classically used to compare decisions under uncertainty (e.g. [6], [18]). However, it has been argued that uncertainty may indirectly modulate behaviour in such designs (see [19] and discussion), and a direct approach is preferred [16]. In this study we focus on a continuous decision-making task in which we require subjects to actively report their estimates of the mean and confidence of uncertain visual stimuli. We will ask the question of how these estimates are formed from the evidence provided, specifically addressing how the visual cues that comprise the stimulus are integrated to form a robust percept of its mean and variance.

To achieve these aims we present a novel experimental paradigm that requires subjects to explicitly track the mean and variance of noise-perturbed visual cues. We control the arrival of noisy visual stimuli over time, allowing us to monitor the behavioural consequences as sensory evidence accumulates. In two variants of our “butterfly catching” task we ask subjects to (i) track the *mean* of “fluttering” visual cues (*viz.* localising a butterfly); and (ii) indicate the *range* in which they believe the mean of the cues to lie (*viz.* choosing an appropriate size of net).

From trial-to-trial we modulate the underlying distribution of the cues, allowing us to observe the evolution of mean and confidence estimates with respect to the visual cues responsible for their formation. Using a sensorimotor model we show the extent to which the observed trajectories are statistically-optimal under the kinematic limitations of human motion, while computation of the weights allocated to each visual cue over time allows us to expose the mechanisms of sensory integration underlying the processes of continuous estimation.

## Results

### Experimental Paradigm

**Figure 1:**
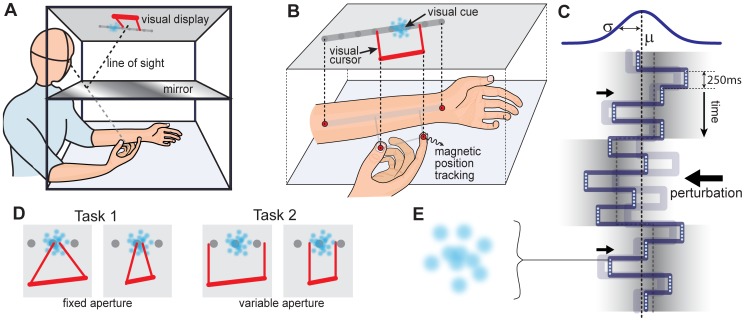
Experiment Setup. Illustration of the *butterfly-catching experiment* setup. (**A**) **Projection Rig.** Subjects placed their left forearm under a mirror, and used their right hand to localise 2D visual stimuli that appeared at a random *target location,* 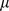, along the forearm. (**B**) **Cursor Control.** Using a mirror aligned with a rear-projection screen we presented visual feedback onto the horizontal plane of the arm. We used a 3D magnetic tracking system to record forearm and finger positions. Finger positions were represented by a 2D visual cursor and the arm by a target line. Visual cues (top half of figure) were aligned veridically with tactile and proprioceptive cues (bottom half of figure). (**C**) **Manipulations.** A total of 15 visual cues were presented in each trial. Each cue, lasting 250 ms, was chosen from an underlying distribution with mean 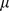 and variance 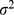. On each trial we randomly varied 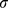 to manipulate the uncertainty of the cue distribution. On each trial we randomly perturbed the mean of one-third of the cues by 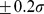 (and shifted the remaining cues by 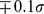, preserving the overall mean). In the figure we show a negative perturbation of the second block, exaggerated in magnitude for illustrative purposes. (**D**) **Tasks.** Subjects performed two tasks: (i) In Task 1, subjects were asked to estimate the *mean* of the stimuli with the position of their right hand, indicated by a fixed-aperture visual cursor; (ii) in Task 2 they were asked to indicate the *range* in which they believed the mean to lie with the spacing of their thumb and forefinger, indicated by a variable aperture visual cursor. (**E**) **Visual Cues.** Each visual cue is composed of a sequence of 5 random dot clouds, one of which is shown for illustration.

In this paper we introduce the “butterfly catching” paradigm, illustrated in figure 1. Subjects are required to judge the statistical properties of a “fluttering” temporal sequence of visual stimuli which are projected onto the line of their left forearm. Subjects localise the stimuli with a variable sized “net”, indicated by lines projected from the forefinger and thumb of their right hand. They are successful in a given trial if the mean of the stimuli lies within the aperture of their net, and are given points at the end of each trial if successful. For a complete description of these details see *Materials and Methods*.

The fluttering visual cues are a sequence of blurry dot-clouds, with cloud locations distributed in time according to a pseudo-Normal distribution with mean 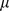 and variance 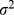 (see figure 1C and *Materials and Methods*). The perceived uncertainty of clusters of noisy visual samples changes as a predictable function of their number [12], but in the present study the noisy clusters are distributed in time rather than space so that we can examine the continuously evolving perception of the mean and uncertainty of the stimuli as evidence arrives over time.

In Task 1 we examine subjects’ ability to estimate the mean, 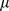, of the visual stimuli using a cursor with small fixed aperture (figure 1D, *left*). We modulate the variance of the visual cues, 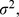
from trial-to-trial. The maximum score is attained when subjects navigate to the true mean of the stimuli.

In Task 2 subjects must instead indicate the range of values in which they believe the mean to lie, using a variable cursor aperture (figure 1D, *right*), with width determined by the distance between the thumb and forefinger. From Task 1 we establish a linear mapping from 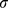 to mean endpoint error to provide performance feedback in Task 2 that forces subjects to report their *objective uncertainty* (by optimising the trade-off between accuracy and point-scoring). We assume subjects can acquire this mapping during the 450 trials preceding Task 2.

Task 2 demands subjects to report their mean and confidence estimates simultaneously, providing a unified paradigm to evaluate the mechanisms underlying the formation of these statistical estimators. To expose these mechanisms we manipulate the distributions of the stimuli from trial-to-trial in two ways: (i) we modulate the variance of the visual cues, 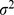; and (ii) we add perturbations to subsets of the cues, (block 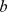, direction 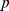). 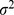, 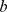 and 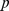 are chosen randomly from trial-to-trial.

In manipulating the cue variance (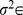 *low*, *medium* and *high*) we hypothesised that subjects would estimate the mean and (based on [15], [20]) report the objective variability in their performance. An increase in cue variance should be reflected in both an increased distribution of errors in localising the mean and decreased confidence.

To induce perturbations we divided the sequence of cues on a given trial into three blocks (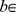 *early*, *middle*, *late*) and shifted cues in a given block by 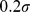 in a chosen direction (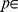 *negative*, *positive, neutral*). All other cues were shifted in the opposite direction by 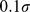, so that the overall mean remained the same. We hypothesised that subjects would integrate the cues over time to compute mean and confidence estimates. By inducing within-trial cue perturbations we can infer the contribution of each cue in the sequence to the final decision.

We found that subjects were equally good at mean estimation in both tasks, shown in *Figure S1*. To compare the two tasks (excluding trials with perturbations) we conducted a within-subjects analysis of variance (ANOVA) on the mean endpoint error (the mean absolute deviation of the final mean estimate from the target), with a two-level factor of task (*Task 1* and *Task 2*) and three-level factor of 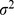 (*low*, *medium*, *high*). This revealed a significant main effect of 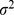 (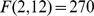, 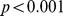) but no main effect of task (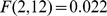, 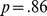) and no interaction (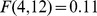, 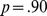). The significant effect of 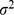 confirms that the variance manipulation increases the task difficulty as expected. The absence of task effect indicates that Task 1 performance variability is a reliable predictor of Task 2 performance variability, justifying the score function used in Task 2.

### Continuous Estimation of the Mean

**Figure 2:**
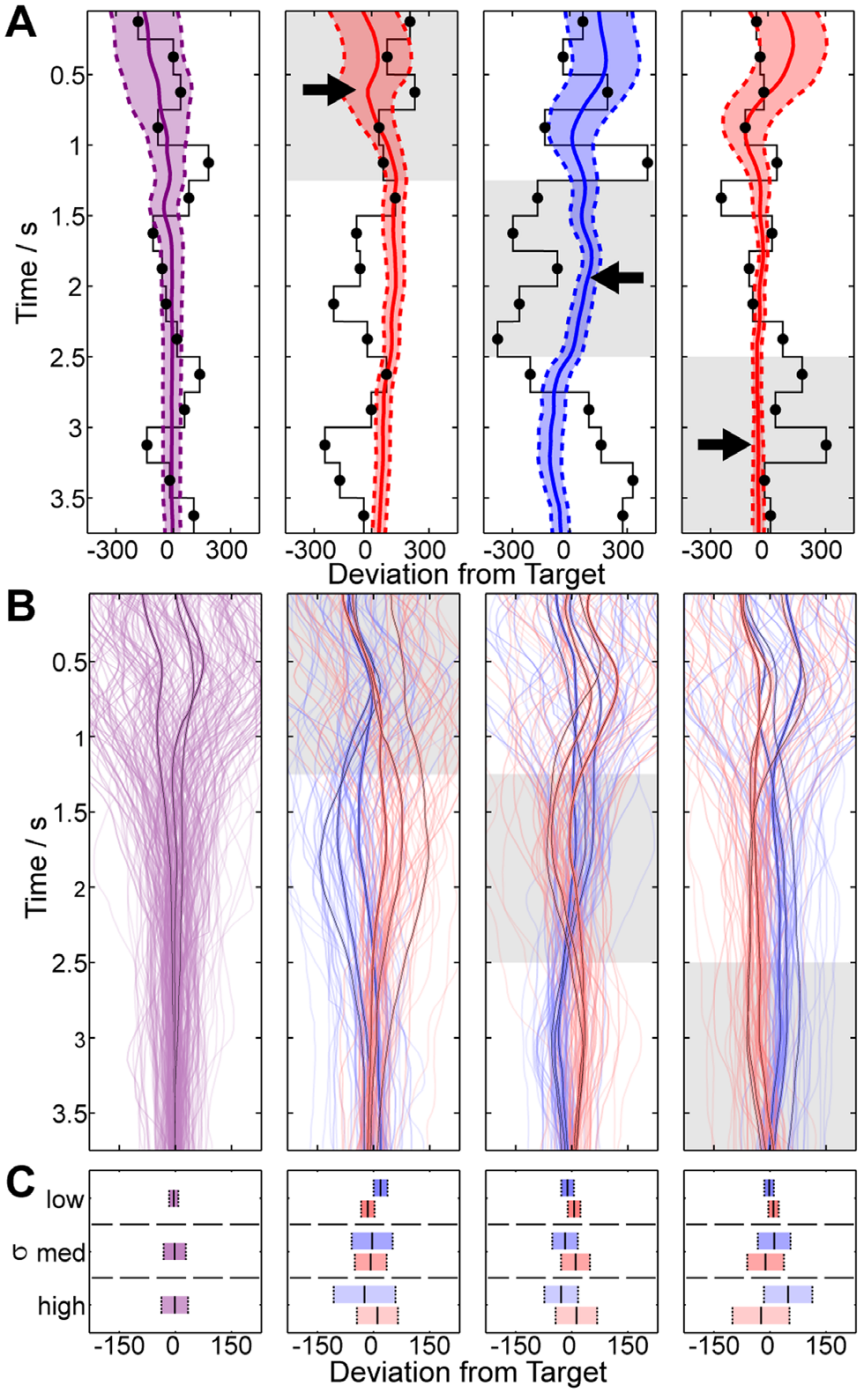
Data for a single subject in Task 2. (**A**) **Typical trajectories** for four experimental conditions. On each trial the subject’s estimates of the mean (solid line) and confidence (dotted line) are affected by the sequence of cues (black dots). From left to right we plot the no perturbation, early, middle and late-onset perturbation conditions. Perturbation of different blocks (shaded and with arrow) results in corresponding trajectory deviations. (**B**) **Average trajectories for one subject.** We plot the average trajectories for one subject for negative (blue), zero (purple) and positive (red) perturbations, for each 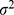. The averages for each condition (darker lines) highlight the main trends. (**C**) **Endpoint Variability.** There is a high level of variability in the trajectories in B, though much of this may be explained by the added variance and perturbations. We plot the mean (solid line) 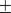 the variance (dotted line) of the *endpoint of the trajectory* for each experimental condition to illustrate this. Late-onset perturbations result in greater endpoint errors and endpoint variability scales with 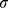.

In figure 2 we present the resulting trajectories for a typical subject performing Task 2. Figure 2A shows four example trajectories which illustrate the consequence of early, middle and late-onset perturbations on decisions. From the smooth trajectories it appears that subjects gradually accumulate sensory evidence, responding (after a delay) to perturbations. Though there is high variability across trials (figure 2C) we observe distinct trajectories for the different experimental manipulations (figure 2B).

**Figure 3:**
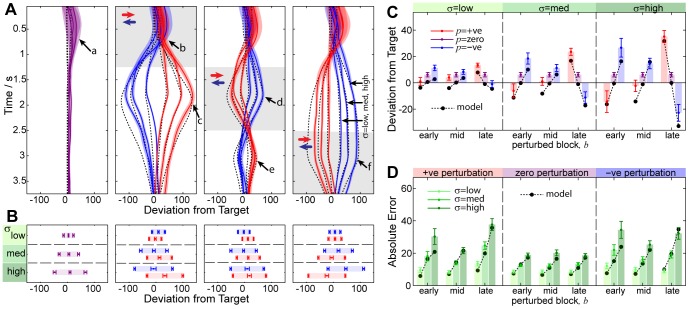
Continuous mean estimation data grouped across subjects. (**A**) **Average Trajectories.** We show the average empirical trajectories across subjects compared to our model predictions. Trajectories are computed for each subject by averaging over all trials for each condition. From left to right we plot the no perturbation, early, middle and late-onset perturbation conditions (shaded). The empirical trajectories for negative (blue), zero (purple) and positive (red) perturbations are plotted for each value of 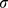 (labelled). Each trajectory shows the mean across subjects 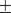 the standard error of the mean (SEM). Key features of the empirical data include cue-induced deviations (arrows b, d and f) and subsequent corrections as further evidence arrives (arrows c and e). Note the qualitative and quantitative nature of the model fit to the data (dashed line). (**B**) **Endpoint mean and variability.** At the end of each trial the position of the cursor represents subjects’ final estimate of the *mean*, and the width of the cursor represents subjects’ final estimate of the *confidence*. For each of the experimental conditions we plot the mean across subjects 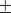 SEM of the left bound of the confidence estimate, the mean estimate and the right bound of the confidence estimate. Subjects show increasing confidence windows for larger values of 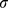 (from top to bottom) and show deviations from the target as a result of the perturbations (red and blue). (**C**) **Endpoint Error.** For each of the experimental conditions we show how the final deviation of the mean from the target is a predictable function of variance 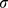, perturbation magnitude 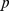 and block 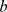. The model makes a reasonable quantitative fit for all conditions, though note that it does not capture the asymmetry in the empirical data (which is slightly positively biased) (**D**) **Absolute Endpoint Error.** The final absolute deviation of the mean from the target captures the average error in the task. This error increases with 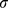 and with perturbations, the magnitude of which is also explained by the model.

In figure 3 we present the results averaged across subjects. The distinguishing features of the empirical trajectories (figure 3A) are (i) high initial variability (arrow *a*); (ii) trajectory deviations shortly after the onset of the perturbation (arrows *b*, *d* and *f*); (iii) spontaneous changes in direction (i.e. inflexions, arrows *c* and *e*); and (iv) endpoint errors (deviations of the final estimate from the target, figures 3B, 3C and 3D); From the interval of the standard error across subjects it is apparent that these phenomena are robust. Note that in figure 3A the trajectories are centred on the true target location (the average of all cues in the sequence, including those which are perturbed). Recall that the perturbation of a given block is balanced by perturbations of half-magnitude of the remaining blocks in order to preserve the overall mean. This results in deviations that oppose the larger perturbation prior to its onset and follow the larger perturbation after its onset (for example, note that that a rightward perturbation in block 3 is balanced by a leftward perturbation of blocks 1 and 2). Responses to perturbations demonstrate the within-trial contribution of cues to perception.

We devised a model of motor behaviour to account for the latencies observed in decisions (see *Materials and Methods*). The model observer integrates the visual cues in a statistically optimal fashion (by computing the maximum likelihood mean estimate). This estimate manifests itself through the movement of the cursor, which we constrain by introducing three parameters, namely sensorimotor latency, 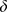, maximum speed, 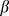 and momentum 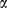 (see *Materials and Methods*). This model accounts both qualitatively and quantitatively for the key features of the empirical data, such as the magnitude and timing of direction changes, and the magnitude of endpoint deviation and endpoint error (figure 3C and 3D). The model parameters were optimised per-subject to ensure the best possible fit to the data (see *Materials and Methods*), but these parameters are global to all conditions and have no capacity to explain the role of individual cues on decisions, nor the effects of cue perturbations or variance (see *Discussion*). In figure 3C we see that the empirical data is biased in the positive direction. For the unperturbed condition, the model predicts an average deviation of zero but the empirical data shows a +6 pixel deviation. It is unlikely that this small systematic error is due to an alignment issue between the visual stimuli and the hand, as the apparatus was carefully calibrated and the effects of visual-spatial mismatch on task performance are expected to be minimal [19]. We suspect that the systematic error may be due to subjects’ preference for certain limb configurations and is an unavoidable consequence of the task. Nevertheless, the timing and magnitude of the key features of the empirical data are accurately predicted by our model. This indicates that subjects can form a continuous estimate of the mean which evolves over time as evidence arrives.

### Mechanisms of Temporal Cue Integration

To understand the mechanisms by which subjects estimate the mean we can infer the contribution of each of the visual cues to the evolving estimates. These are computed per-subject by linearly regressing the cue locations to the decision made at each time-step, over all trials (for full details see *Materials and Methods*).

**Figure 4:**
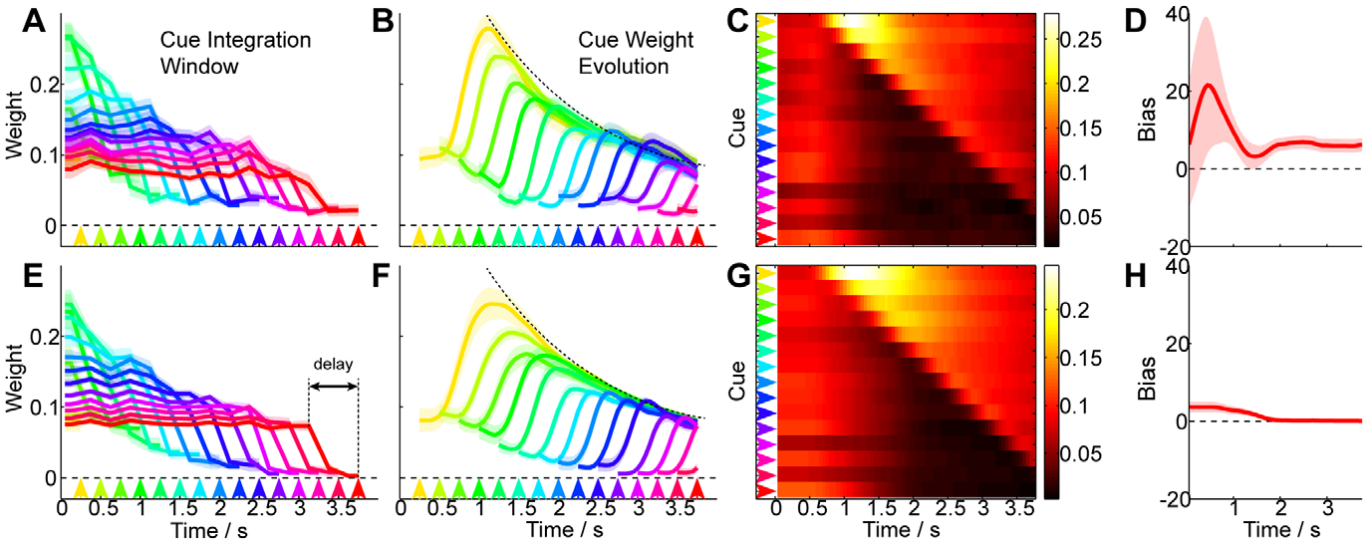
Mean Estimation Cue Weight Evolution. To measure the evolution of weights assigned to each visual cue we perform a linear regression of the position of each cue in the sequence to the measured trajectory, using data over all trajectories for each subject (see *Materials and Methods*). In this figure we illustrate the match between the empirically observed weights and the model predictions. (**A**) **Empirical Data Integration Window.** At each time-step in the trial we infer the weight assigned to each cue in the sequence. These weights define a window of cue integration which changes over time as evidence arrives. We plot the weights assigned to the cues seen so far (solid lines) 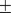 SEM across subjects (shaded), omitting weights assigned to future cues for clarity (but see C and main text). Coloured arrows indicate the time-step at which the corresponding integration window applies. At all time steps we see that the observed cues are given approximately *equal* weight, with the exception of a 0.5 s time lag. This weight equality is indicative of optimal integration (as we see in E). (**B**) **Empirical Data Cue Evolution.** In an alternative visualisation of A we plot the weight allocated to each cue (solid line) 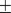 SEM (shaded) as it evolves over the time-course of a trial. Each curve corresponds to the cue arising at the time marked by the corresponding coloured arrow. For clarity we do not show the weight allocated to the cue prior to it being seen (but see C and main text). This plot reveals that shortly after being seen, each cue’s weight suddenly increases as it contributes to the estimate, settling at a weight that is the same across all cues. These weight profiles are indicative of optimal integration (as we see in F). (**C**) **Empirical Weights.** The weight matrix 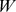, excluding the systematic component, captures the evolution of cue weights over time (see *Materials and Methods*). When visualised in this way, using colour to represent cue weight, we can see the initial response delay and the evolution of cue combination, as summarised in A and B. The regression method can not establish the cause of the initial 0.5 seconds of the trajectory, indicated by equal weights assigned to all cues (including future cues). This weight matrix is indicative of optimal integration (as we see for the optimal matrix 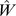 in G) (**D**) **Empirical Systematic Bias.** In computing the regression of cue to decision we allow for a systematic component to capture the variability in the trajectory that is not explained by the cue weights. We observe empirically a non-zero systematic bias in the positive direction, especially for early time steps. Our optimal model predicts the initial bias (as we see in H), but the overall bias observed is sub-optimal. We believe this to be an unavoidable consequence of the configuration of the experiment (see text) (**E-F**) **Model Predictions** for comparison, with three parameters (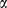, 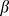 and 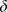) optimised to minimise the difference between 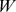 and 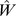 (plots C and G).

Figure 4 shows the resultant cue weights for the empirical trajectories (figures 4A–4D) and the model trajectories (4E–4H). Our regression method assigns a weight to each cue (including cues that have not yet been observed), quantifying its contribution to the decision at each time step. The weight assigned to future cues provides useful validation that the regression method is successfully discriminating the contributions of each cue and not fitting noise. During the initial 0.5 s of the trajectory we see that causality can *not* be reliably discerned, and therefore all cues (including future ones) are equally weighted (figure 4C). However, after this brief initial stage we see that the weight assigned to future cues declines, indicating that empirical decisions are correctly attributed to only the observed cues.

In figure 4A we plot the “integration window” at different times within the trial - this illustrates theweights assigned to all of the the observed cues at each time-step. We notice that each line is approximately horizontal, indicating that each cue contributes equal weight to the decision at each time step. In figure 4B we plot a curve for each cue to show how each cue’s weight rises after it has been seen, then gradually decays as more evidence arrives to share equal weight with the other cues. This can be visualised in *Video S1*.

The systematic component of the weight regression (figure 4D) reveals an initial bias of +20 pixels, but this subsides after 1 second. The large initial variability is due to the randomisation of the target location 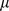, which subjects quickly navigate towards. A slight positive bias of around +6 pixels remains for the entire trajectory, which is also observed in trajectory data (figure 3C). The weight regression confirms that this is not a cue-driven error but indeed a systematic error.

We use the same regression method to plot the weight matrix for the ideal-observer model subject to kinematic constraints (see *Materials and Methods*). We find a close qualitative and quantitative match (figure 4E–4H), except that the model does not reveal an overall systematic bias.

### Continuous Estimation of the Uncertainty

**Figure 5:**
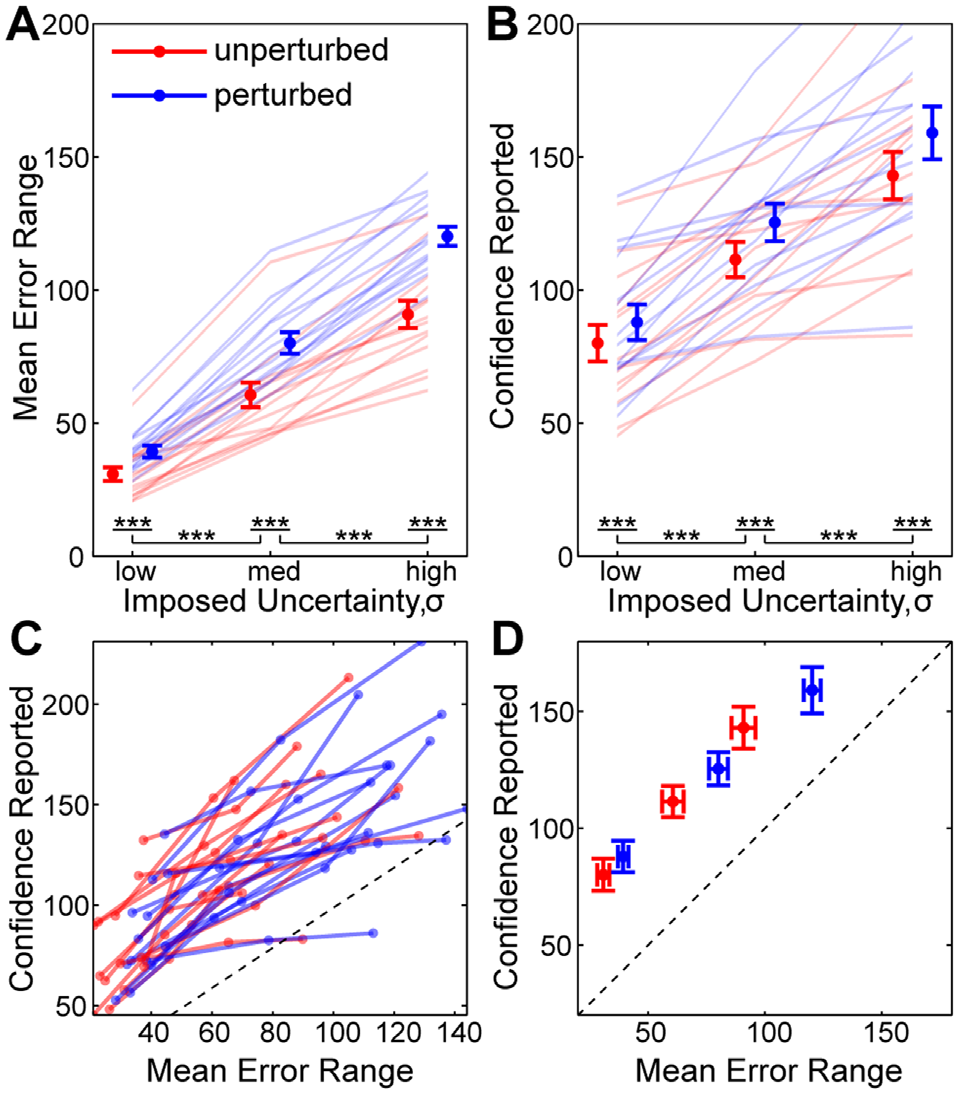
Uncertainty Estimation Performance. In this figure we show that subjects are able to discern the different levels of uncertainty added to the cues. (**A**) **Objective Uncertainty.** We plot the mean error range (twice the mean absolute deviation of the final mean estimate) 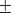 SEM, for different levels of 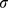 (solid blobs and error bars), for perturbed (red) and unperturbed (blue) trials. In addition we overlay the average results for each subject (faded lines). Subjects show statistically significantly increased errors as a result of both cue uncertainty and the presence of perturbations. Between-subject variability is low, as indicated by the distinct separation between red and blue lines and the consistency of the gradient. (**B**) **Subjective Uncertainty.** We plot the average width of subject’s confidence window at the end of the trial for each 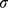 and perturbation, similar to A. Subjects show a statistically significantly increased confidence window as a result of both cue uncertainty and the presence of perturbations, mimicking the objective uncertainty. However, between-subject variability is high, indicating that different subjects have widely differing abilities at estimating uncertainty. (**C**) **Subjective-Objective Mapping.** We combine per-subject data from A and B, plotting the mean error for each condition versus the confidence reported. The ideal mapping is shown by the dotted line. Subjects consistently over-estimate the objective uncertainty. (**D**) **Grouped Subjective-Objective Mapping.** We plot the average mapping across subjects 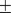 the SEM in each direction. This demonstrates the consistency with which subjects over-estimate their objective uncertainty.

Thus far we have analysed continuous mean estimation behaviour. In this section we analyse subjects’ ability to estimate sensory *uncertainty.* . In figure 5 we compare the *objective error range*, equal to twice the mean absolute error (equation 3, in *Materials and Methods*), to the reported (*subjective*) confidence window. These quantities are identical for the ideal-observer.

To assess the effect of task manipulations (objective uncertainty), we conducted an ANOVA on the objective error range with within-subject factors of perturbation (*unperturbed* vs *perturbed*, grouping over the perturbation conditions) and 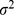 (*low*, *medium* and *high*). This revealed a significant main effect of 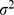 (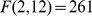, 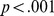), a significant main effect of perturbation (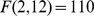, 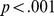), as well as a significant interaction between 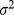 and perturbation (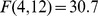, 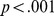). The interaction was expected since the perturbation magnitude is a fraction of 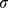.

To assess the subjective effect of task manipulations (perception of uncertainty), we also conducted an ANOVA on the confidence window range, with within-subject factors of perturbation (*unperturbed* vs *perturbed*, grouping over the perturbation conditions) and 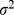 (*low*, *medium* and *high*). This revealed a significant main effect of 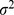 (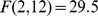, 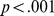), a significant main effect of perturbation (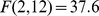, 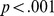), as well as a near-significant interaction between 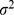 and perturbation (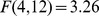, 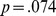). The magnitude of the interaction was less than expected.

The ANOVA results above indicate that the task manipulations have significant behavioural consequences, modulating both the objective uncertainty as well as perception of this uncertainty. We conducted t-tests to compute the differences between conditions, and found that unperturbed trials resulted in fewer errors than perturbed trials (measure: *objective error range*, 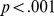 for all 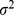) which was reflected in increased confidence (measure: *confidence window*, 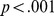 for all 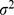). Likewise, the increase in error for 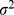 between low to medium and medium to high conditions (measure: *mean error range*, 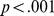 for both perturbation conditions) were reflected by reduced confidence (measure: *confidence window*, 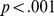 for both perturbation conditions). Figures 5A and 5B provide a graphical representation of these findings.

We consolidated figures 5A and 5B to examine the relationship between objective variability and subjective perception. In figure 5C we show the results per-subject, and see from the positive slope of each line that subjects were able to discriminate the level of sensory uncertainty in each condition, although with much variability across subjects. 96% of the data lies above the line 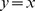, indicating that subjects’ confidence windows consistently over-estimate the objective variability. In figure 5D we show the average data across subjects.

**Figure 6:**
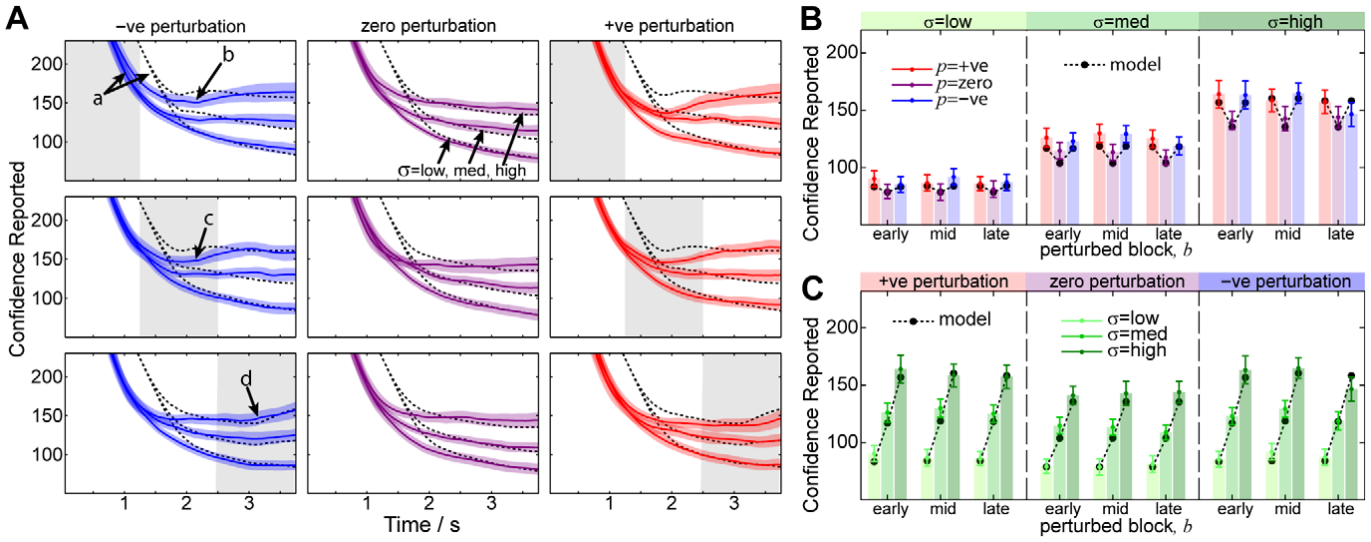
Continuous uncertainty estimation data grouped across subjects. This figure illustrates the quantitative match between the model and the data. (**A**) **Average Trajectories.** In this figure we show the average empirical trajectories across subjects compared to model predictions. Trajectories are computed for each subject by averaging over the trials for each condition. From top-to-bottom we plot the early, middle and late-onset perturbation conditions (indicated by shaded region), and from left-to-right we plot negative (blue), zero (purple) and positive (red) perturbation directions. The resultant trajectory for each 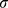 (labelled) shows the mean across subjects 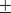 SEM. The model fit to the data is shown using a dashed line. Note that the model does not explain the initial part of the trajectory (arrow *a*), but does reasonably well at explaining the timing of deviations in uncertainty perception that arise as a consequence of perturbations (arrows *b*, *c* and *d*) (**B and C**) **Confidence Reported.** For each of the experiment conditions we show how the endpoint subjective uncertainty is a predictable function of variance 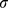, perturbation magnitude 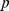 and block 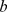. We plot the same results grouped in different ways for comparison. The model makes a good quantitative fit for all conditions, but note that the model contains a systematic *safety margin* parameter 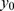 which may explain some aspects of the data fit (see text).

We have seen above that subjective confidence can reliably discriminate perturbation-induced and variance-induced objective uncertainty at the end of the trial. This behaviour also holds for continuous confidence perception. In figure 6A we plot the average confidence estimate trajectories across subjects. The distinguishing features of the empirical trajectories are (i) trajectories are indistinguishable for the first 0.5 seconds, but then diverge; (ii) low, medium and high variance result in correspondingly-scaled confidence windows after divergence; (iii) sudden increases (inflexions) in confidence window occur as a result of early-, middle- and late-onset perturbations (arrows *b, c* and *d*); and (iv) final decisions vary with variance and perturbation onset (6B and 6C). From the interval of the standard error across subjects it is apparent that these phenomena are robust.

**Figure 7:**
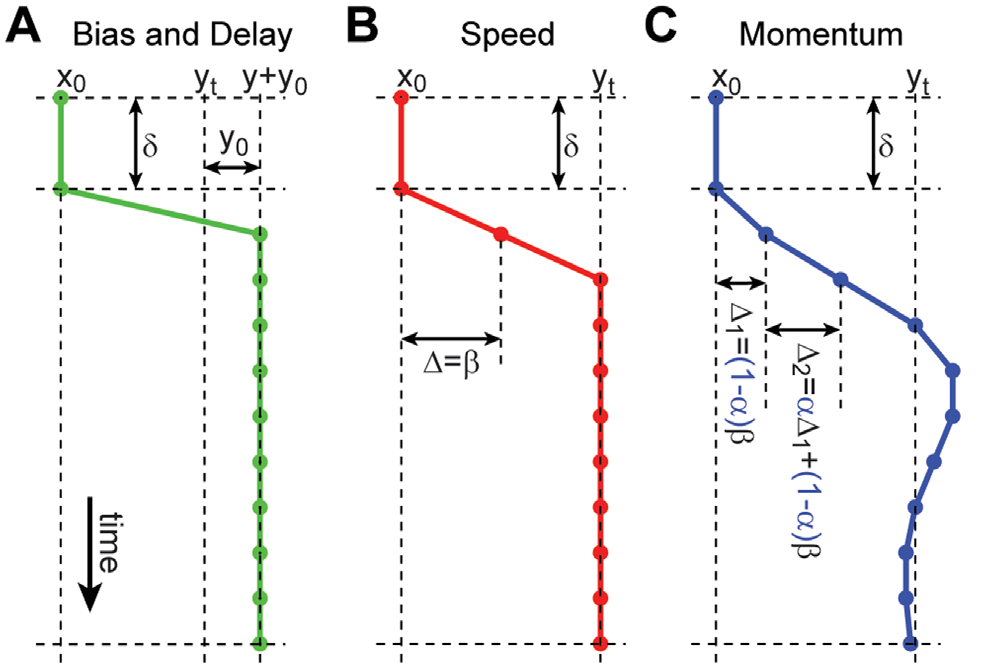
Model of Sensorimotor Kinematics. In order to explain subject’s evolving trajectories over time we model the inevitable kinematic constraints on movement. In the model we assume that, other than these limitations, subjects will behave as ideal observers. We discretise movement into 50 ms time-steps. At time-step 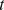, for an observer aiming to reach a target 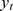 they make a displacement of 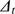, moving them from position 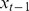 to 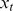. This figure illustrates the parameters of the model. (**A**) **Bias and Delay.** We assume that there is some delay, 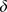, before subjects initiate their movement. This captures sensory, processing and motor delays. Subjects may also have some inherent bias in one direction or another, due to the configuration of the experiment or otherwise, so we introduce a bias parameter 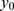. (**B**) **Speed Constraint.** We assign a maximum speed, 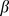, to limit the displacement in a given time step. (**C**) **Momentum Constraint.** We assume that subjects can not accelerate instantaneously by introducing a smoothing parameter 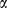 on 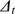.

We devised a kinematic model to account for these observations (see *Materials and Methods* and figure 7). The modelled observer optimally integrates the deviations of cues from the current mean estimate so as to maximise the expected reward (which is achieved when the confidence window equals one standard deviation of the objective uncertainty either side of the sample mean). Similar to the previous analysis for mean estimation, we maintain the three parameters of sensorimotor latency, 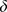, maximum speed, 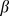 and momentum 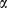. Owing to the consistent over-estimation of uncertainty discussed previously, we include an additional safety margin parameter, 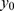.

This model accounts both qualitatively and quantitatively for the key features of the empirical data, such as the magnitude and shape of variance-induced differences, the magnitude and timing of perturbation-induced inflexions, and the magnitude of the final decision for each condition. The per-subject model parameters were optimised to ensure the best possible fit to the data, but nevertheless have no capacity to explain the within-condition effects of perturbations or variance. While the safety margin parameter 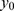 does have the capacity to explain the overall magnitude of decisions, it is simply a per-subject constant and can not explain the differences between the trajectories (see *Discussion*).

It is interesting to note that the increase in perceived uncertainty resulting from cue perturbations (figure 6A, arrows *b*, *c* and *d*), occur at the same time as mean estimation changes of direction (figure 3A arrows *c*, *d* and *g*). The mean estimation and uncertainty estimation tasks appear to be coupled, though our model treats them separately. This could explain the initial discrepancy between our model and the data (figure 6A, arrow *a*): presumably subjects do not adjust their confidence window until they have first navigated toward the target (after about 1 second). The model provides a good fit to the remainder of the trajectory.

In computing the weight matrix to explain the evolution of cue weights (see *Materials and Methods*) we find that the empirical weights do not reflect optimal performance (see *Figure S3*). We see empirically that each cue deviation contributes to the final decision, but the resultant weight profiles are noisy and difficult to interpret. This may indicate that subjects are sub-optimal at estimating uncertainty from time-evolving visual cues (but see *Discussion* for alternative interpretations).

## Discussion

We have shown that subjects estimate the mean of time-varying stimuli in a predictable manner. By manipulating the variance as well as the onset and direction of perturbations we have shown that this estimate is computed in a statistically-principled way that assigns equal weight to all observed cues to form a final estimate. We devised an ideal-observer model that is subjected to kinematic constraints. We find a close match between the empirical data and our statistically-optimal model, suggesting that subjects can accumulate evidence over time to form *optimal continuous estimates* of the mean of noisy visual stimuli.

By manipulating the variance of the underlying stimuli we examined the relationship between *objective uncertainty* and *subjective uncertainty*, showing that the two are closely, but not directly coupled. By manipulating subsets of the cues through perturbations we also evaluated the respective weighting given to each cue for confidence estimation, and showed that, with the addition of a conservative *safety-margin,* we can reliably predict responses to cue variance and perturbations. While the evolution of cue weights was not well explained by our model, possibly indicative of sub-optimal integration, subjects were clearly capable of accumulating evidence over time to continuously discriminate different levels of uncertainty due to to cue variance and cue perturbations.

In making decisions, subjects must make a trade-off between allocating time to perception, and time to action [2]. Since there is a considerable time delay between sensing the world and initiating motor actions, subjects often make decisions while sensory information is arriving. Discrete events (such as subjects “changing their mind”) may be based on the time-delayed accumulation of evidence [21]. In this paper we show how subjects form decisions based on visual cues and update their estimate as evidence arrives, as indicated by deviations in trajectories under different levels of perturbation. In our continuous task these inflexions are not discrete “changes of mind” but in fact continuous decisions related to the subject’s evolving perception of uncertainty.

The approach presented in this paper utilises a continuous time-varying task, providing a window into the processes of mean and uncertainty acquisition. The modulation of uncertainty in alternative designs, such as the two-interval forced-choice paradigm, may induce “apprehension” in proportion to the imposed uncertainty [19], which may indirectly provide a measure of stimulus uncertainty that does not require an explicit representation of uncertainty [19]. Experimental manipulations to increase uncertainty, such as decreasing stimulus contrast or adding uncorrelated noise, may increase the latency with which subjects can react to stimuli, again providing interpretations absent of explicit uncertainty awareness. Even our method of time-varying jittering cues may trigger mechanisms that could indirectly account for uncertainty judgements. It has therefore been argued that much research on statistical optimality includes situations in which an *implicit* internal representation of uncertainty may explain task performance (see [19] and e.g. [5], [6], [10], [18], [22], [23]). However, by asking subjects to report their uncertainty one can directly tackle the question of whether subjects can *explicitly* acquire representations of sensory uncertainty, applicable to reaching tasks [16], numerical estimation tasks [24] and visual perception tasks [15]. In this paper we have extended this idea further to consider the *continuous* estimation of uncertainty as evidence arrives.

To what extent are the observed continuous trajectories optimal? The global parameters of the model are optimised to achieve the best fit for each subject, but as these parameters are fixed across all trials they can not explain the differences in the trajectories observed for each condition - these can only be explained by the contribution of individual cues to the decisions (although the parameters can explain the general shape of the trajectories and the latency after which cues contribute to the trajectories). In the mean estimation model the cue contributions are chosen optimally (i.e. according to the ML estimate of the mean). The resultant close match between the empirical and observed trajectories for each of the conditions indicates optimal cue weighting. In contrast, in the confidence estimation model a suboptimal “safety margin” is used to explain the magnitude of the estimate and thus a match between empirical and model trajectories does not indicate optimality. This safety margin causes subjects to significantly over-estimate uncertainty, resulting in less than optimal performance in the task.

Could the finding of optimal mean estimation and suboptimal confidence estimation be explained by subjects relying on a simpler heuristic? For example, subjects may position their thumb and forefinger on the extremes of the cues seen so far, or choose an aperture size proportional to this range. This was our primary motivation for computing the weights assigned to each cue in the sequence, which revealed that each cue was approximately equally weighted for the mean-estimation task. This would not be the case for subjects relying on subsets of the cues: as the mean of the cues is not equal to the median due to perturbations, the suboptimal heuristic strategies would result in different endpoint decisions, different trajectories and different weight profiles. We therefore posit that mean estimation trajectories are indeed based on optimal cue weighting. In contrast, uncertainty estimation empirical weights do not match the optimal model weights. The presence of a consistent overestimation of uncertainty indicates that subjects may be relying on a subset of the observed cues to form their estimate. Nonetheless, subjects still increase their aperture in response to uncertainty increases and perturbations, indicating that subjects do have access to some measure of their objective uncertainty.

A number of studies have observed underconfidence in forced-choice tasks (e.g. see [15], [25]), consistent with the present finding of subjective overestimation of objective uncertainty. In a recent study in which subjects were asked to report a confidence window when predicting the magnitude of random samples from a time-varying distribution, subjects showed perceptual biases when estimating the uncertainty [24]. This was attributed to a pre-learned bias and was otherwise consistent with a Bayesian observer model, although could equally be explained by an inability to accurately gauge the magnitude of the uncertainty, as in the present study.

In addition to the possibility of suboptimal uncertainty estimation, from the present results there are a number of alternative potential causes of over-estimated uncertainty: (i) It is not known if subjects fixate on the jittering stimuli or on the cursor, which may effect their ability to accurately judge (or anticipate) the stimulus location (see [26]); (ii) Subjects may not have been able to maximise their expected gain (in contrast to [27]), due to differences in experimental design; (iii) The kinematic model fit to the data may be insufficient to describe behaviour; (iv) The data collected may have been too noisy for reliable model fitting. To address points (i) and (ii) further research is needed to decouple the factors that determine objective variability and performance maximisation. For example, subjects were not aware of the exact functional form of the score function (in contrast to [27]) adding additional learning demands. Whilst the effects of learning were not observed in the data these potential limitations of the scoring system should be noted. To address points (iii) and (iv) we must evaluate the viability of our kinematic model (See *Materials and Methods*, and figure 7). In our model the delay parameter captures the combined effect of sensory and motor latency and motor kinematic limitations are captured by speed and momentum parameters, which affect the overall shape of the trajectories. It was found that these three parameters were sufficient to explain the average empirical data for mean estimation. Alternative models may introduce additional parameters to explain different aspects of the data, such as the addition of sensory and motor noise or separate sensory and motor delays. Further experiments would be required to test such models.

Our experiment design utilised a grasping task within a fixed plane. As the task does not abstract the cursor or targets to a computer screen, it maintains many aspects of ordinary grasping (visual feedback, proprioceptive feedback, feedforward control etc.), keeping the task as natural as possible. As detailed in the methods, feedback of the fingers was aligned with the true finger locations (see [22]). The design relied on the fact that subjects could independently control their grasp aperture and hand position, which we felt was likely (although see [28]; independent finger and thumb control has not been conclusively demonstrated). Target stimuli were presented along the line of the left forearm, though it could also have been achieved by presenting stimuli along any fixed line in the plane. We chose to use the arm as a reference because (i) this design lends itself to a number of follow-up experiments in which the cues may be tactile rather than visual; and (ii) it allows subjects to position both the target line (with their left arm) and the cursor (with their right arm) in any comfortable configuration of their choosing.

Our results are consistent with a number of studies that report optimal multisensory integration, (e.g. audio-visual [7], [18], visuo-haptic [6], [19], [22] visuo-proprioceptive [29] and visual [4], [30] integration). However, these results provide *indirect* evidence of subjective representation of objective uncertainty [15]. In the present study we find that subjects are able to form an optimal estimate of the mean and an overestimate of the uncertainty, providing *direct* evidence of continuous mean- and confidence-estimation mechanisms that may underlie the observation of optimal integration. In contrast, there are a number of studies in which optimal behaviour was not observed. Multisensory integration studies have demonstrated a significant under-weighting of sensory uncertainty for texture information [10] and auditory information [13] and in a third study it was found that visuo-haptic integration performance was inconsistent with maximum likelihood estimation in more than 80% of the data [11]. However, the authors conceded that subjects may have attempted to combine cues optimally but did not have an accurate estimate of the variance of the individual cues. Consistent with this finding, in the present study we have observed a suboptimal *safety-margin* in subjects estimating their uncertainty. By extending our experimental paradigm to multiple sensory modalities we would predict different integration weights for subjects using either *subjective* or *objective* uncertainty to form multimodal estimates. By allowing for simultaneous measurement of mean and confidence our experimental paradigm readily lends itself to the testing of such hypotheses.

There is a growing body of research which aims to understand the neural substrate of uncertainty representation. For example, neural firing activity in orbitofrontal cortex in rats is an accurate predictor of olfactory discrimination uncertainty [17], and neurons in parietal cortex encode information about the degree of decision-making uncertainty in monkeys [31]. The presence of confidence-estimation mechanisms in the brain is supported by biologically plausible computational models (such as reviewed in [32]) in which neural populations readily encode sensory uncertainty and allow networks to compute posterior probability distributions. The results presented in this paper provide direct evidence that humans have rapid and reliable access to statistical information available from stimuli, which could presumably be attained from such neural representations.

### Conclusion

Our quantitative paradigm allows us to simultaneously measure mean and confidence estimation ability. It allows us to observe these processes over time as we control the arrival of evidence. We are able to make qualitative and quantitative predictions of the performance of subjects based on a statistically optimal model constrained only by elementary kinematic limitations. The paradigm naturally lends itself to a wide variety of future experimental manipulations, for example in understanding the methods deployed when integrating cues from multiple modalities, for understanding the time-courses of decisions, and for decoupling the roles of objective and subjective uncertainty perception for decision-making.

## Materials and Methods

### Experimental Methodology

#### Subjects and ethics

14 volunteers participated in this experimental study. All subjects were healthy, right-handed and aged between 21 and 30. All of the subjects were naive to the experimental manipulations and the experiment apparatus. The experimental protocols were in accordance with the University of Edinburgh School of Informatics policy statement on the use of humans in experiments. Subjects gave informed consent before participation in the study and received financial compensation for their time (approximately 90 minutes per subject).

#### Apparatus

Subjects were instructed to place their left forearm under a horizontal mirror onto an array of tactile markers, serving as a tactile reference frame consistent and veridical with the visual display. Using the rear-projection mirror setup as illustrated in figure 1A, visual feedback was given in the plane of the arm so that feedback of the arm and finger locations aligned with the true finger and arm locations, removing any confounding effects of mismatch between visual and proprioceptive cues (as discussed in [23]). The use of the left arm as a reference frame allowed subjects to position themselves comfortably. Further, this setup lends itself naturally to an alternative version of the task in which stimuli are tactile rather than visual (see *Discussion*).

Stimuli were anti-aliased and projected using a high resolution video projector with latency 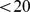
ms. 1 projected pixel corresponded to approximately 0.3 mm on the arm.

To enable accurate 3-D tracking of the arm and fingertips we used a Polhemus Liberty 240 Hz 8-sensor motion tracking system (POLHEMUS, USA). Every 50 ms we sampled the arm and fingertip positions and logged data using custom personal computer (PC) software. The same PC software was responsible for displaying and logging the stimuli, ensuring that our data and stimuli were temporally calibrated.

#### Task 1: mean estimation

In Task 1 subjects were instructed to indicate the *mean* of a sequence of visual stimuli, using a fixed-aperture cursor (figure 1D, *left*). The cursor location was computed as the mean of the orthogonal projections of the thumb and forefinger position vectors onto the forearm.

Each subject underwent an initial training period to become familiar with the task (phase *1A*), followed by a block of trials to assess mean estimation performance as we varied the visual uncertainty, 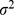, from trial-to-trial (phase *1B*).

#### Task 2: mean and confidence estimation

In Task 2 subjects were instructed to indicate the *range* in which they believed the mean to lie, using a variable aperture cursor (figure 1D, *right*) determined by orthogonal projections onto the arm of their thumb and forefinger. The average position of the projections was interpreted as their mean estimate and the range as their confidence in this estimate.

Again, each subject underwent an initial training period to familiarise them with the task (phase *2A*), followed by a larger block of trials to assess their combined mean and uncertainty estimation performance as we varied 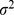, 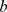 and 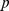 from trial-to-trial (phase *2B*).

#### Task manipulations

We manipulated the distributions of the stimuli from trial-to-trial in two ways: (i) we modulated the variance of the visual cues (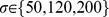 pixels, which we term *low*, *medium* and *high* uncertainty respectively); and (ii) we added perturbations (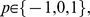 termed *negative*, *neutral* and *positive* respectively) to subsets of the cues (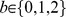, termed *early*, *middle* and *late* respectively). Table 1 summarises the use of these manipulations.

**Table 1:** Experiment Structure.

		Structure		Configuration				
4–5 7–10 Task	Phase	Sessions	Trials	*N_σ_*	*N_b_*	*N_p_*	*N_r_*	Total
1	A	3	15	3	55	55	0	135
1	B	3	15	3	55	55	0	135
2	A	4	15	3	55	55	0	180
2	B	1	15	3	3	3	135	540

Each subject performed 990 trials in total across four experimental phases. Task 1 examined subjects’ ability to estimate the *mean* of a jittering visual cursor, split into a training phase (*1A*) and a test phase (*1B*). In Task 2 we examined the subject’s ability to report their *confidence* in this estimate in addition to reporting the mean, again with a training phase (*2A*) and a test phase (*2B*). Subjects performed several sessions in each phase to improve data integrity. On each trial we presented 15 cues, distributed pseudo-randomly with variance 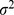, and split the trial into blocks, perturbing a given block 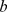 in direction 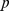. We examined 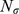, 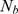 and 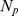 levels of each of these manipulations respectively, listed in the table. We also included 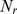 trials of random duration (between 5 and 15 cues in length). For each configuration subjects performed 15 trials. All sessions and trials were randomly shuffled within a phase.

In order to interpret the subtle effects of these manipulations robustly we used sets of pseudo-random cue sequences which were counterbalanced across 15 trials for each manipulation (see *Visual Stimuli*). Subjects completed several sessions each with different sets of cue sequences. The order of all trials and sessions were randomised, and on every trial the target location, 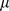, was chosen at random.

In Task 2 we also added trials of shorter durations (durations randomly chosen in the range 5 to 15 cues). One-sixth of trial were of this nature, but these trials did not contribute to our analyses. They were included to ensure subjects would not be able to predict when each trial was going to end, encouraging continuous behaviour.

#### Performance feedback

In Task 1, 10 points were awarded if the trial was successful. To motivate subjects in Task 2, subjects were awarded less points if their chosen confidence interval was greater than the *expected objective uncertainty* determined in Task 1, encouraging them to estimate and report their objective uncertainty. We exploit the finding that subjects can learn to maximise expected reward [27].

On a given trial 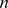 let the measured cursor position and width be given by be 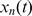 and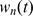 respectively, recorded over the trial duration (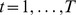). On completion of a trial we assume 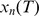 represents a subject’s internal estimate of the mean, 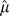, and 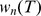 their internal estimate of the confidence interval, 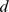. We use a score function, 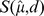, which assigns a score according to success or failure:
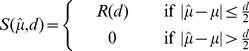
(1)Where successful trials are rewarded according to



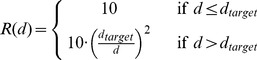
(2)The reward function penalises apertures larger than 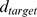. In our experiment, 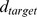 is calculated for each subject based on the data empirically observed in experiment phase *1B*. We first compute the *objective error* as the mean absolute endpoint deviation for each 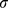, denoted by 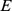:
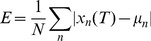
(3)


We then define an *objective error function* for each subject, 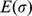, determined by the linear mapping between 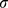 and 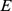. On a given trial in Task 2 we compute the standard deviation of the cues, 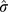, and use the objective error function to determine 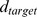, twice the expected objective error:
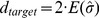
(4)


The target aperture size for the confidence estimation task could have been chosen to be any quantity proportional to the objective variability. Regardless of the choice of target aperture size, subjects are required to learn the mapping from stimulus to confidence interval in order to succeed at the task. It was an assumption of our approach that this could be done so as to maximise the expected score (as per [27]). We decided to set to the target aperture size to be the range of values that form approximately one standard deviation of the objective variability on either side of the mean.

If subjects pick an aperture smaller than 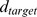 this decreases the probability of success, while an aperture larger than 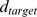 decreases the score. The reward function in equation 2 ensures that the overall maximum expected reward is achieved by choosing an aperture of exactly 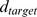. This method, therefore, encourages subjects to estimate their own *objective error range.* Further details can be found in [33].

#### Visual stimuli


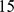
 visual cues are presented in each trial. For mathematical convenience we describe the visual cues as a sequence of 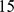 locations 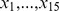, where each 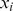 is drawn from an underlying distribution with mean 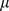 and variance 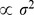. Each visual cue is presented for 250 ms.

Each visual cue comprises 5 frames. On each frame for the 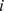
th cue we generate a cloud of ten random blobs distributed with a standard deviation of 10 pixels in horizontal and vertical directions and centred at 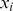. Each blob is a low-contrast 2-D Gaussian of radius 8 pixels (based on [18]). Blob-clouds provide a way to modulate the underlying difficulty of the task, but in this experiment we did not modulate the cloud parameters.

Each 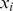 is chosen according to shuffled pseudo-Normal cue sequence 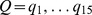 (generated by taking uniformly-spaced samples from the inverse cumulative Normal distribution, then shuffled). We devised an algorithm (illustrated in *Figure S2* and described in further detail in *Text S1*) to generate a matrix of cue indices 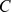, with 15 columns (one for each trial, 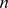) and 15 rows (one for each cue 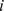). Each entry of the matrix 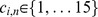 is an index into 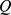, shuffled by our algorithm so as to maximise the unpredictability of each trial while removing uncontrolled sources of uncertainty.

To generate each 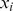 on a given trial 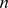 we use 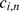 as an index into 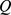, then add spatial uncertainty by multiplying by 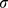 and induce perturbations by shifting the mean of one third of the cues by 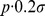 and the remaining cues by 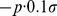. We vary 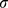, 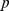 and 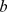, and the random target location, 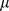, for each trial. Hence, we have
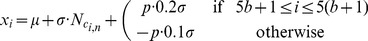
(5)


This is repeated using the same 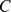 and 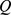 for all experimental configurations. Subjects complete multiple sessions for each phase of the experiment using some or all of the above manipulations as previously discussed. Each session uses different instantiations of 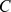 and 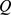, and all sessions within each phase of the experiment are shuffled. Each subject receives different instantiations of 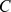 and 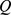.

### Data Analysis

#### The ideal observer

During a trial, as samples accumulate we would expect an *ideal observer* to accurately estimate the sample mean and sample variance of the cues thus far seen and make decisions based on this available evidence. Given 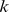 cues 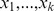 the unbiased sample mean and sample variance are given by equations 6 and 7:
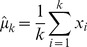
(6)

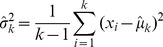
(7)


In Task 1 the observer’s ideal strategy is to select 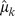 at time 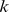.

One can show that the variance of the sample mean estimator is given by
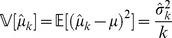
(8)


Thus, in Task 2 the ideal observer strategy at time 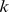 is to select a confidence interval equal to 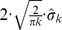, which is equal to the ideal-observer objective error range (as described in *Performance Feedback*).

#### Sensorimotor delay model

The ideal observer can perform instantaneous computations and act on sensory information immediately, but human beings can not. In the presence of inevitable sensory, processing and motor delays and noise we consider how the ideal observer would now perform. We define an ideal-observer model constrained by the three global parameters, 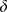, 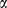 and 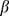, capturing natural kinematic constraints on hand motion.

Suppose the observer has witnessed 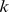 cues by time 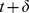 due to sensory delays. We introduce modified estimates of mean and variance from equations 6 and 7:
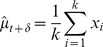
(9)

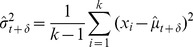
(10)


In Task 1 subjects can compute 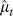 from equation 9 to form a time-delayed internal estimate of the mean.

In Task 2 we expect subjects to estimate their objective uncertainty. From equation 10 the ideal-observer can calculate the time-delayed variance estimate 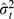, which is translated into an objective error range 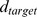 (using the linear *objective error function* 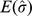 defined previously; see equation 4) to achieve the maximum possible score.

In addition to sensory delays we introduce motion constraints. At time 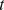 let us define the *reported estimate* (i.e. the position of the cursor) as 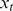, and the *perceived estimate* (i.e. our time-delayed internal estimate of the mean) as 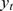. In our formulation we model the observer as making discrete steps of size 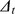 so that the reported estimate smoothly converges to the perceived estimate. The model constrains motion using two parameters: a maximum speed parameter, 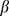, constrains the maximum displacement made by the observer in a given time-step; and a momentum parameter, 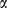, prevents sudden speed changes by smoothing these displacements over time. i.e.
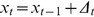
(11)

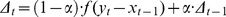
(12)

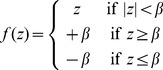
(13)


Note that the model applies to both mean and confidence judgements: For Task 1 we set 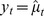, and for Task 2 we replace 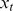 with 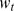 (the width of the cursor at time 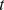) and set 
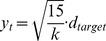
.

We add one additional parameter to the confidence estimation model, a *bias* term, 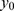. In equation 12 this replaces the term 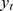 with 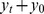. This can be thought of as a safety margin or constant systematic error. This is considered a suboptimal component of the model, while the other parameters capture natural kinematic limitations.

Figure 7 illustrates the effect of each of these parameters on model trajectories.

#### Weight regression

To compute the contribution of each cue in the trial to the empirical trajectory observed we perform a multiple linear regression at each time-step using the non-negative weight least-squares algorithm described in [34]. For full details see *Text S2*.

For Task 1 we regress the pixel *location* of the cues in the trial (including those not yet seen), plus an additional constant, to the *position* of the cursor at that time.

For Task 2 we regress the *absolute deviation* of the pixel locations of the cues in the trial from the sample mean, plus an additional constant, to the *width* of the cursor at that time.

#### Model parameter learning

Our model (see *Sensorimotor Delay Model*) has relatively few parameters. We optimise these parameters to achieve the best fit to the data, but note that this process does not confound our claims. The model does not modify the magnitude of weights assigned to each cue, it merely constrains the trajectory through which a decision manifests itself.

Using the same weight regression technique for the model data we compute a parametrised weight matrix 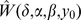. For full details see *Text S2*. We minimise the square of the difference between 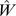 and the empirical 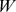 with respect to the model parameters using the constrained interior-reflective Newton minimisation method described in [35], [36], implemented in Matlab (Mathworks Inc., USA). To improve the rate of convergence we normalise the systematic weight terms 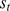 prior to minimisation, to compensate for their excessive magnitude relative to the cue weights.

For the mean estimation model we set 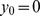 and do not allow for its optimisation. This sub-optimal term is not necessary to explain the gross features of the data.

## Supporting Information

Figure S1 **Overall Task Performance.** In this figure we show the final absolute deviation of the cursor from the target location for different levels of uncertainty in Task 1 and Task 2. Trials with perturbations are excluded. Note that both tasks give indistinguishable mean-estimation performance, indicating that ability at Task 2 is not compromised by the additional demands of the task. We posit that Task 1 performance is a good indicator of task 2 performance.
(TIF)

Figure S2 **Pseudo-Random Cue Sequence Generation.** In this figure we illustrate the Saundoku Algorithm for generating pseudo-random cue sequences. The purpose of this method is to ensure that cues are counterbalanced across trials so as to minimise systematic biases to the data, while at the same time presenting no additional information to subjects to aid their success at the task. **(A) Cue generation.** The sequence of cues to be used for a trial are generated from a pseudo-Normal distribution, created by sampling the Inverse cumulative Normal distribution function at equally spaced intervals (red blobs). The output (black blobs) is distributed *pseudo-Normally*, i.e. as the number of samples increases the histogram of the samples converges on the Normal probability density function. These samples are shuffled (blue blobs) to provide a cue sequence. The method of shuffling is illustrated in sub-plots B-E. **(B) Initial Cues.** We create a square *shuffle matrix* with rows for cue number (in time) and columns for trial number. Each matrix entry corresponds to a cue generated in sub-plot A. We initialise the matrix with diagonals as shown to ensure that each cue appears only once in each trial, and once in every trial. In the figure for clarity we show 60 cues per trial and therefore 60 trials per condition, but in practice we have only 15 cues per trial and 15 trials per condition. **(C) Trial Shuffle.** We randomise the order of trials to reduce the correlation between neighbouring trials. This does not violate the constraint that each cue appears only once in each sequence, and in every trial. **(D) Partial Cue shuffle.** We then randomise the order of cues within each trial, but we limit the shuffling to within the first, second and final third of the sequence. This maintains the constraint that each cue appears only once in each sequence, and in every trial, and adds the additional constraint that each third contains all cues an equal number of times. **(E) Random Seed.** Finally, each entry of the matrix indexes into the shuffled pseudo-Normal sequence in sub-plot A. The resulting plot appears completely random, but we know the correlations between trials, and we know the average mean and variance for the first, second and third block of trials across all trials.
(TIF)

Figure S3
**Confidence-Estimation Model Weights.** To measure the evolution of cue weights we perform a linear regression of the deviation of each cue in the sequence from the current mean estimate to the confidence window width, using data over all trajectories (see main text *Materials and Methods*). In this figure we illustrate the poor match between the empirically observed weights and the model predictions. **(A) Empirical Data Integration Windows.** At different time-steps in the trial (indicated by coloured arrows) we compute the weight allocated to all cues in the sequence (coloured curves) 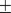 the SEM across subjects. The weights assigned to future cues are not shown. This plot reveals that the decision at each time step is due to a weighted average of the cues deviations observed until that point. These weight profiles do not match the model (as we see in E) **(B) Empirical Data Cue Evolution.** An alternative visualisation of cue weight evolution shows how the weight allocated to the cues at each of the time steps evolves over the time-course of a trial. We do not show the weight allocated to the cue prior to it being seen. This plot reveals that, shortly after being seen, each cue’s weight increases as it contributes to the estimate, then gradually decays. These weight profiles do not match the model and rise much more slowly (as we see in F). **(C) Empirical Weights.** The weight matrix 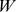, excluding the systematic component, captures the evolution of cue weights over time (see main text *Materials and Methods*). When visualised in this way, using colour to represent cue weight, we can see the initial response delay and the evolution of cue combination, as summarised in A and B. This weight matrix only roughly matches the model (as we see in the plot of 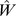 in G), but the high level of noise makes it difficult to reliably fit the model to the data. **(D) Empirical Systematic Bias.** In computing the regression of cue to decision we allow for a systematic component to capture the variability in the trajectory that is not explained by the cue weights. Our model roughly predicts the shape of the systematic component **(E-F) Model Predictions** for comparison, with four parameters (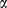, 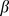, 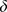 and 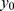) optimised to minimise the difference between 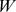 and 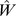 (plots C and G).(TIF)

Text S1 **Shuffled pseudo-Normal cue sequence generation.** Further details of the cue sequence generation process.
(PDF)

Text S2 **Cue weight regression algorithm.** Further details of the method used to compute the contribution of each cue in the trial to the empirical trajectory observed.
(PDF)

Video S1 **Video showing the evolution of the weights contributing to the mean estimate in Task 2.** The contribution of each weight forms an integration window which changes as evidence arrives. Note that at the final time step the integration window is flat for both the empirical data and the model, indicative of optimal integration weights.
(AVI)
