# Supplementary figures and images for: Continuous Evolution of Statistical Estimators for Optimal Decision-Making
Source: PLoS One. 2012 Jun 25;7(6):e37547. doi: 10.1371/journal.pone.0037547 (PMC3382620; doi:10.1371/journal.pone.0037547)

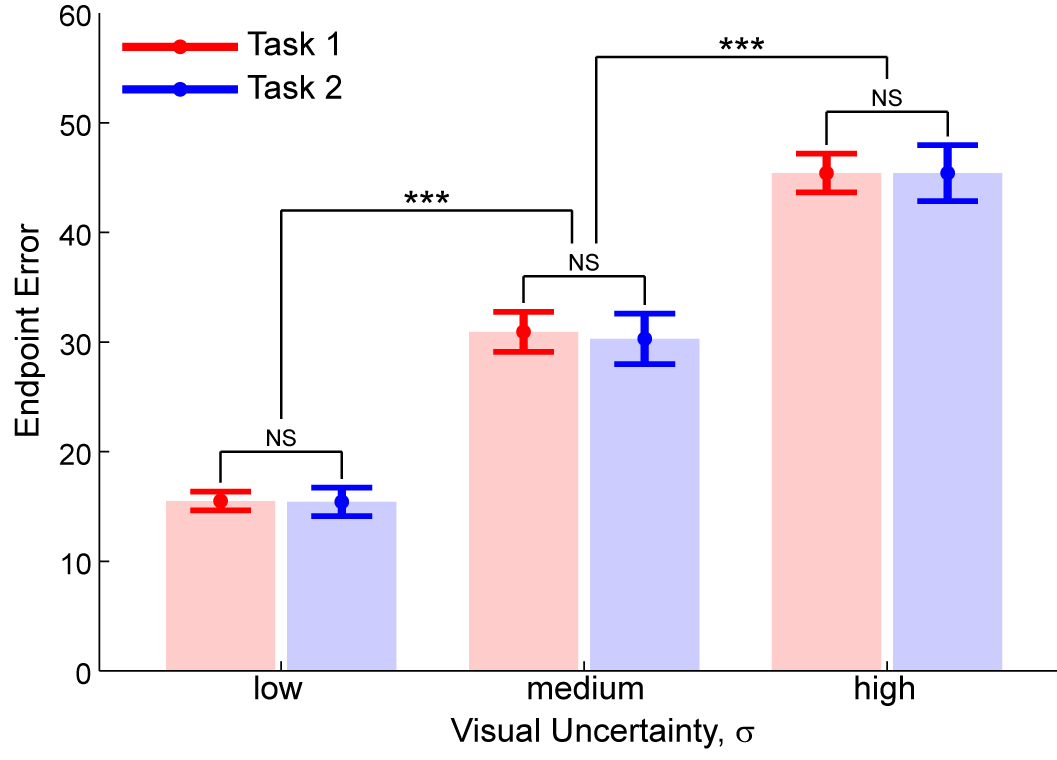

Supplement: Figure S1 — Overall Task Performance. In this figure we show the final absolute deviation of the cursor from the target location for different levels of uncertainty in Task 1 and Task 2. Trials with perturbations are excluded. Note that both tasks give indistinguishable mean-estimation performance, indicating that ability at Task 2 is not compromised by the additional demands of the task. We posit that Task 1 performance is a good indicator of task 2 performance. (TIF) [file pone.0037547.s001.tif]
