# Supplementary material for: Continuous Evolution of Statistical Estimators for Optimal Decision-Making
Source: PLoS One. 2012 Jun 25;7(6):e37547. doi: 10.1371/journal.pone.0037547 (PMC3382620; doi:10.1371/journal.pone.0037547)
Supplement: Text S1 — Shuffled pseudo-Normal cue sequence generation. Further details of the cue sequence generation process. (PDF) [file pone.0037547.s004.pdf]

Supplementary Text S1

Each visual cue x_i is chosen according to a pseudo-random sequence, using the algorithm illustrated in Supplementary Figure S2. The algorithm generates a block of 15 trials, where each trial, n ,contains a sequence of 15 cues. A matrix of cue indices C, with columns for trials and rows for cues for dictates the order cues on a trial, with each entry $c_{i,n} \in \{1, \dots 15\}$. The algorithm is designed to maximise the unpredictability of each trial while maintaining the following constraints:

(i) each cue index appears once and only once in each trial,

$$\forall i, n, j \;.\; i \neq j \implies c_{i,n} \neq c_{j,n} \tag{1}$$

(ii) each cue appears at each time t once and only once across trials within the block,

$$\forall i, n, m \;.\; n \neq m \implies c_{i,n} \neq c_{i,m} \tag{2}$$

(iii) The mean of each third of the trials, averaged over all trials, is exactly zero,

$$\sum_{n} \sum_{b=0}^{2} \sum_{i=5b+1}^{5(b+1)} c_{i,n} = 0 \tag{3}$$

(iv) the variance of each third of the trials, averaged over all trials, is constant,

$$\frac{1}{15} \sum_{n} \frac{1}{3} \sum_{b=0}^{2} \frac{1}{5} \sum_{i=5b+1}^{5(b+1)} \left(c_{i,n} - \sum_{j=5b+1}^{5(b+1)} (c_{j,n}) \right)^2 = 1 \tag{4}$$

These constraints were designed to enable effective data analysis without imposing *uncontrolled* sources of uncertainty. By using this method each cue has equal weight and should contribute equally (on average) to subject's decisions. We then added *controlled* uncertainty to the sequences, as described in the main text.
