# Supplementary material for: Continuous Evolution of Statistical Estimators for Optimal Decision-Making
Source: PLoS One. 2012 Jun 25;7(6):e37547. doi: 10.1371/journal.pone.0037547 (PMC3382620; doi:10.1371/journal.pone.0037547)
Supplement: Text S2 — Cue weight regression algorithm. Further details of the method used to compute the contribution of each cue in the trial to the empirical trajectory observed. (PDF) [file pone.0037547.s005.pdf]

Supplementary Text S2

On trial n let the sequence of cues $x_1^n, \ldots x_{15}^n$ result in the trajectory $Y_n = [y^n(1), \ldots, y^n(T)]^\dagger$ taken by the subject. To compute the weights assigned to the cues over all such trajectories we form a matrix, C, which contains columns for each of the N trials. Each column contains a constant term and the K visual cues for the corresponding trial:

$$C = \begin{bmatrix} 1, & \ldots & 1 \\ c_1^1, & \ldots & c_1^N \\ \vdots & \ddots & \vdots \\ c_K^1, & \ldots & c_K^N \end{bmatrix}$$

We also form a trajectory matrix Y, which also contains columns for each of the N trials. Each column contains the recorded trajectory for the corresponding trial:

$$Y = [Y_1, \ldots, Y_N] = \begin{bmatrix} y^1(1), & \ldots & y^N(1) \\ \vdots & \ddots & \vdots \\ y^1(T), & \ldots & y^N(T) \end{bmatrix}$$

The purpose of the regression is to compute a weight matrix W that minimises the error in the mapping $Y = WC$, where

$$W = \begin{bmatrix} s_1, & w_1(1), & \ldots & w_K(1) \\ \vdots & \vdots & \ddots & \vdots \\ s_T, & w_1(T), & \ldots & w_K(T) \end{bmatrix}$$

We perform the regression described in [1] in a row-wise manner, i.e. for each time-step t.

The resulting weight matrix W can be decomposed into a systematic trajectory $s_{1:T}$ which captures systematic bias not explained by the cues, and a time-series of cue weights indicating the contribution of each cue over time to the trajectory.

For the Task 1 we initialise the cue matrix C with with pixel value of the cue relative to the target location, i.e.

$$c_k^n \quad = \quad x_k^n - \frac{1}{K}\sum_{i=1}^{K} x_i^n \tag{1}$$

We similarly initialise Y with the pixel value of the subject's estimate of the mean centred on the target location.

For Task 2 we initialise C with the *absolute deviation* of the cue from the mean of the cues seen so far, i.e.

$$c_k^n = \left| x_k^n - \frac{1}{k}\sum_{i=1}^{k} x_i^n \right| \tag{2}$$

and we initialise Y with the pixel value of the width of the subject's confidence window.

In performing the regression we allow the systematic component to take any value, but restrict the cue weights to be strictly greater than zero. Since weighting a cue is equivalent to negatively weighting all other cues (due to correlations between cues), this makes the algorithm more stable.

This method also applies to the model trajectories. To generate weights for a parametrised model trajectory we compute:

$$\hat{W}(\delta, \alpha, \beta, y_0) = \begin{bmatrix} \hat{s}_1, & \hat{w}_1(1), & \ldots & w_K(1) \\ \vdots & \vdots & \ddots & \vdots \\ \hat{s}_T, & \hat{w}_1(T), & \ldots & \hat{w}_K(T) \end{bmatrix}$$

We minimise the square of the difference between $\hat{W}$ and the empirical W with respect to the model parameters using the constrained interior-reflective Newton minimisation method described in [2, 3], implemented in Matlab (Mathworks Inc., USA). To improve the rate of convergence we normalise the systematic weight terms s_t prior to minimisation, to compensate for their excessive magnitude relative to the cue weights.

References

[1] C. Lawson and R. Hanson, *Solving least squares problems.* Prentice-Hall, 1974. Chapter 23, p.161.

[2] T. Coleman and Y. Li, "On the convergence of reflective newton methods for large-scale nonlinear minimization subject to bounds," *Mathematical Programming*, vol. 67, no. 2, pp. 189–224, 1994.

[3] T. Coleman and Y. Li, "An interior, trust region approach for nonlinear minimization subject to bounds," *SIAM Journal on Optimization*, vol. 6, pp. 418–445, 1996.
